# Supplementary material for: Lymphatic endothelial cells prime naïve CD8+ T cells into memory cells under steady-state conditions
Source: Nat Commun. 2020 Jan 27;11:538. doi: 10.1038/s41467-019-14127-9 (PMC6985113; doi:10.1038/s41467-019-14127-9)
Supplement: Supplementary file 1 — Supplementary Information [file 41467_2019_14127_MOESM1_ESM.pdf]

SUPPLEMENTARY INFORMATION

Lymphatic endothelial cells prime naïve CD8$^+$ T cells into memory-like cells under steady-state conditions

E Vokali, SS Yu, et al.

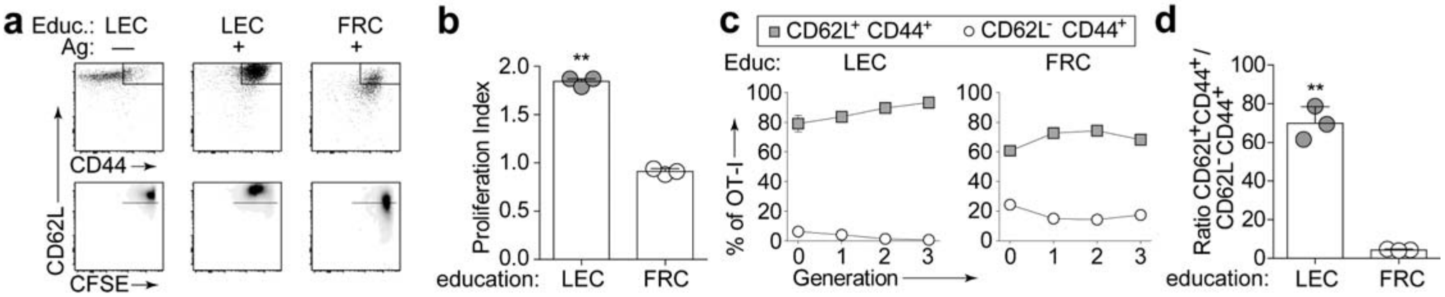

Supplementary Figure 1. Lymphatic endothelial cells (LEC), but not fibroblastic reticular cells (FRC), drive CD8$^+$ T cell proliferation and acquisition of a central memory-like phenotype. Naïve CFSE-labeled OT-I cells were educated for 3 days in the presence of SIINFEKL peptide (1 nM) with LEC or FRC isolated from skin-draining LNs of naïve, healthy WT mice. **a** Representative flow cytometry plots gated on live OT-I cells, showing their expression of CD62L versus the activation marker CD44 (top) or proliferation via dilution of the CFSE dye (bottom). OT-I cells co-cultured with LECs but no antigen are also shown as a control (left). **b** Proliferation index was calculated as the average number of cell divisions that have occurred for any cell that had diluted CFSE. **c** Percentage of OT-I cells that have acquired memory (gray) or effector phenotype (white) as a function of cell divisions/generation. **d** Ratio of memory OT-I cells to effector-like OT-I cells detected depending on antigen-presenting cell type. Mean±SD for n = 3 technical replicates taken from one representative of three independent experiments. $**p < 0.01$ via Student's t-test.

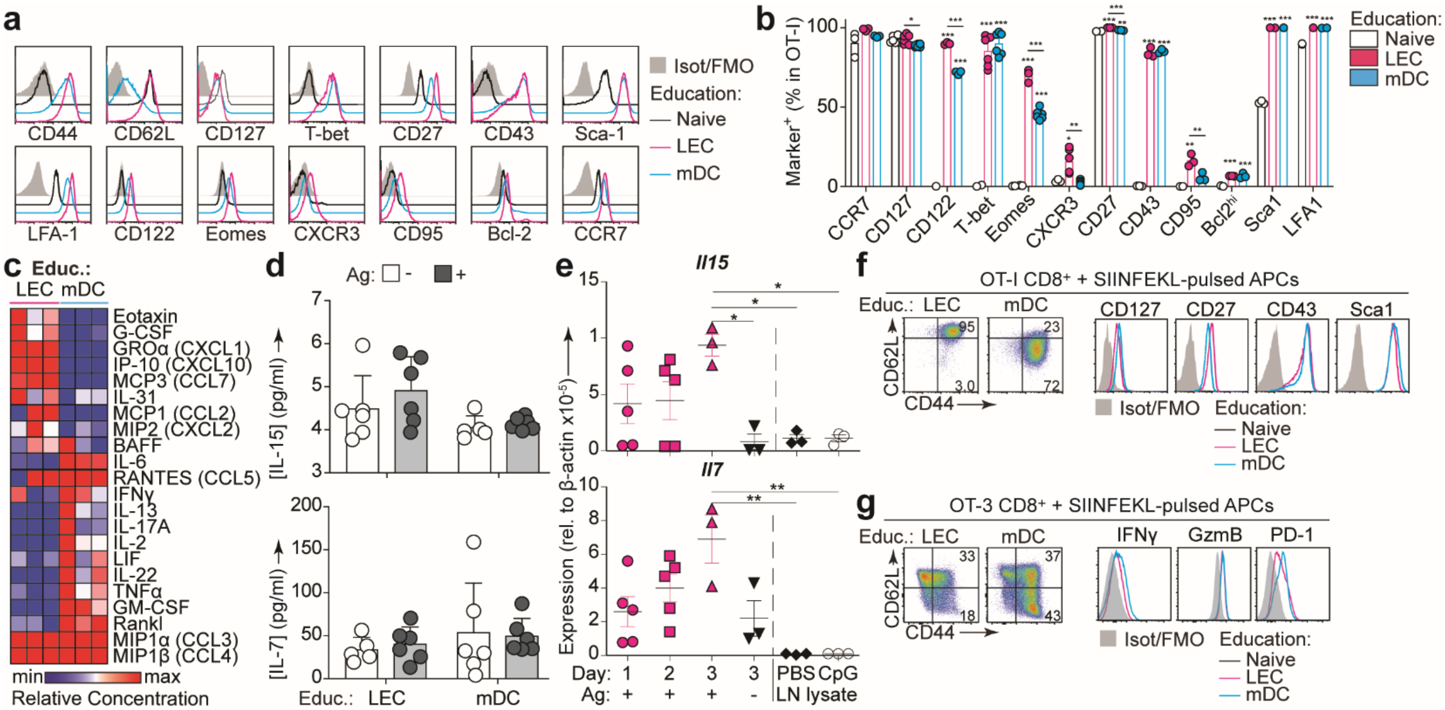

Supplementary Figure 2. LEC-educated CD8+ T cells are phenotypically distinct from mDC-educated cells in expression of markers of central/stem cell memory. a-e Naïve CFSE-labeled OT-I cells were educated in the presence of antigen with LEC or mDC for 3 days unless otherwise noted, or incubated alone (educ: naive). **a** Representative histograms of expression of indicated markers at endpoint, and quantified in **b** among control (mostly naïve CD44-CD62L+), LEC-educated (mostly CD44+CD62L+), or mDC-educated (mostly CD44+CD62L-) OT-I cells. **c** Heatmap depicting relative production of chemokines and cytokines detected in co-culture supernatants, as determined by a multiplex cytokine assay. The color key illustrates production levels relative to control OT-I cells, with blue depicting downregulation and red depicting upregulation. **d** Levels of secreted IL-15 and IL-7 (pg/ml) quantified by ELISA. **e** Quantitative RT-PCR analysis of *Il15* and *Il7* expression, normalized to β-actin as a control housekeeping gene, in LECs over time of co-culture, versus in the skin-draining LNs (LN lysate) of control (PBS) or CpG-treated mice. **f-g** Phenotypic evaluation of OVA-reactive transgenic TCR CD8+ T cell systems following 3d co-culture with LEC or mDC pre-pulsed for 6-8h with 10nM SIINFEKL. (left plots) Representative dot plots of CD44 and CD62L expression gated on live **f** OT-I cells or **g** OT-3 cells, with inset numbers indicated percentages of the indicated T cells that fall within the gates. (righ) Representative histograms of selected memory/effector-related phenotypic markers. Bars and columns depict mean ± SD pooled from up to two of three independent experiments (n=3-6). **p≤0.01, ***≤0.001 by one-way ANOVA followed by Bonferroni's post-test.

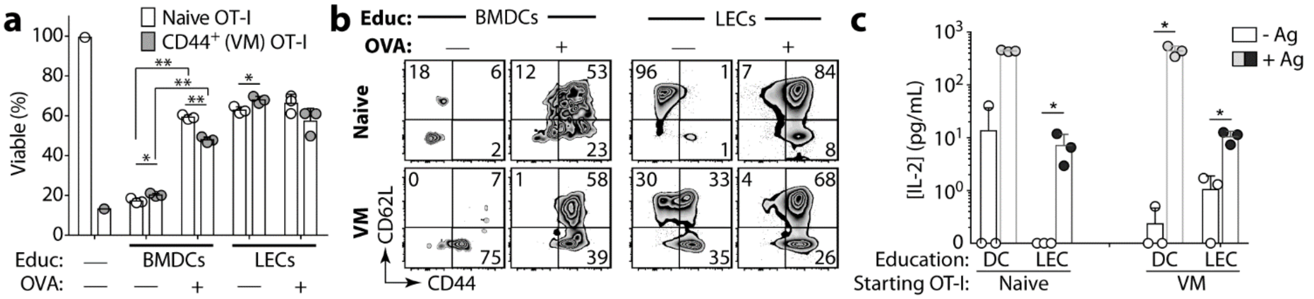

Supplementary Figure 3. Lymphatic endothelial cells induce memory-like phenotype in naïve but not CD44⁺ OT-I cells. CFSE-labeled OT-I cells from a healthy, unchallenged OT-I mouse were sorted based on $CD44^{-}CD62L^{+}$ (Naive) or $CD44^{+}$ (virtual memory; VM) phenotype, prior to 3d co-culture with primary lymph node-derived lymphatic endothelial cells (LECs) or mature bone marrow-derived dendritic cells (BMDCs). For some conditions, LECs and BMDCs were pulsed with 23µM OVA (1 mg/mL) for 6h and washed prior to addition of sorted OT-I cells. **a** Viability of OT-I cells, quantified based on percentage of $CD8^{+}$ $CFSE^{+}$ cells that excluded DAPI. **b** Representative flow cytometry contour plots gated on live OT-I cells, depicting CD44 and CD62L expression and the gating scheme for quantification in Fig. 4k-l. **c** Endpoint concentration of IL-2 in the co-culture supernatants. *$p < 0.05$, **< 0.01, ***< 0.001 by two-way ANOVA with Bonferroni post-test. n = 3 taken from one representative of two independent experiments (For resting, naïve OT-I cells, n = 1 was shown for comparison but excluded from statistical analyses). For b inset numbers indicate frequency of live OT-I cells within the gate.

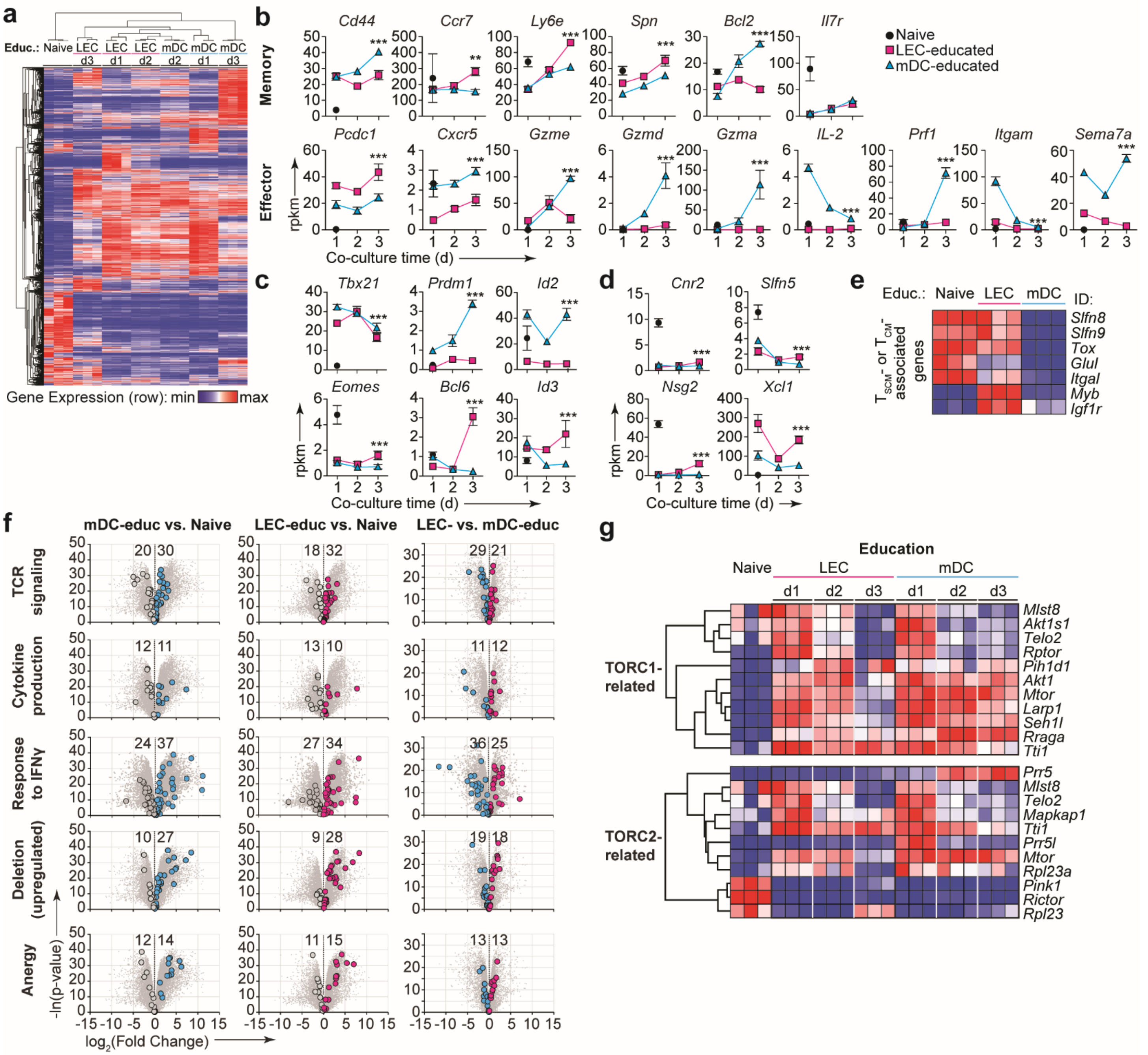

Supplementary Figure 4. Differential expression analysis of transcriptomes of LEC- versus mDC-educated CD8⁺ T cells. Naïve OT-I cells were cultured alone (Educ: Naïve) or educated in the presence of 1nM antigen by LEC or mDC for the indicated time before transcriptomic analysis by RNA sequencing. **a** Hierarchical clustering analysis of genes which were most affected by LEC/mDC education relative to uneducated/naïve OT-I cells. **b** Normalized relative expression levels (rpkm, reads per kilobase per million mapped reads) of genes linked to memory- (top) or effector (bottom) phenotype. **c** Relative expression levels of genes encoding transcription factors promoting $CD8^+$ T cell effector (*Tbx21, Prdm1, Id2*) versus memory (*Eomes, Bcl6, Id3*) differentiation **d** Relative expression levels of genes encoding proteins associated with late-memory[46]. **e** Heatmap of selected genes reported to be differentially expressed among T_{SCM} and T_{CM} cells.[33] **f** P airwise differential expression analysis of naïve, LEC-educated, or mDC-educated OT-I in the form of volcano plots, with each row of plots highlighting genes linked to the indicated biological functions as annotated by the Gene Ontology Atlas.[47] **g** Heatmap displaying the relative gene expression levels for genes associated with mTORC1 or mTORC2 signaling, as annotated by the Gene Ontology Consortium. Data points and error bars indicate mean ± SD of n = 3 biological replicates. Columns within heatmaps reflect data from individual biological replicates. *p≤0.05, **≤0.01, ***≤0.001 for LEC- vs. mDC d3 education by one-way ANOVA with Benjamini-Hochberg correction for FDR.

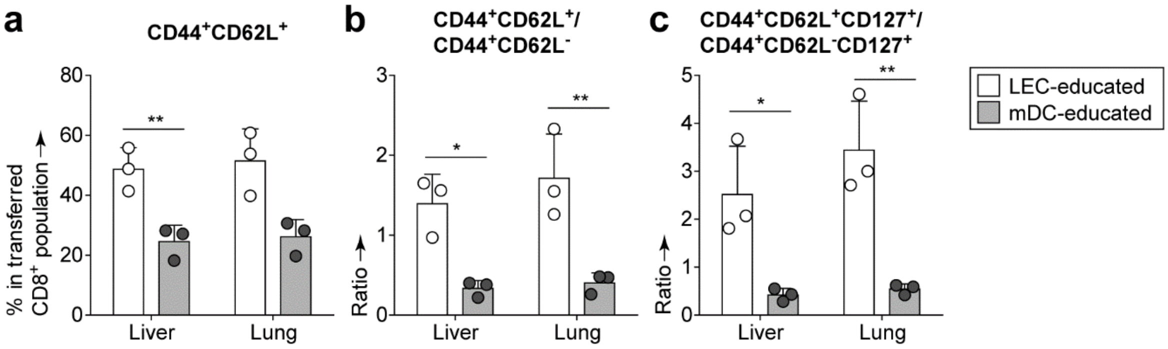

Supplementary Figure 5. LEC-educated $CD8^+$ T cells retain central memory-like phenotype after *in vivo* transfer, even after migration into peripheral organs. Experimental schedule as in **Fig 6a**. $CD45.1^+$ OT-I cells were educated in the presence of antigen (1nM) by LEC or mDC, transferred i.v. into healthy WT C57Bl/6 mice (10^6 cells/recipient), and phenotypically analyzed by flow cytometry 1w post-transfer. **a** Percentage of LEC- or mDC-educated OT-I cells recovered in the liver and lung that expressed $CD44^+CD62L^+$ phenotype. **b** Ratio of $CD44^+CD62L^+$ OT-I cells over $CD44^+CD62L^-$ OT-I cells; and **c** ratio of T_{CM} ($CD44^+CD127^+CD62L^+$) OT-I cells over T_{EM} ($CD44^+CD127^+CD62L^+$) OT-I cells as a function of their initial education conditions and the organ wherein they were recovered. Data points and error bars depict mean ± SD for n = 3 biological replicates from one representative of two independent experiments. *p≤0.05, **≤0.01 by two-way ANOVA with Bonferroni's post-test.

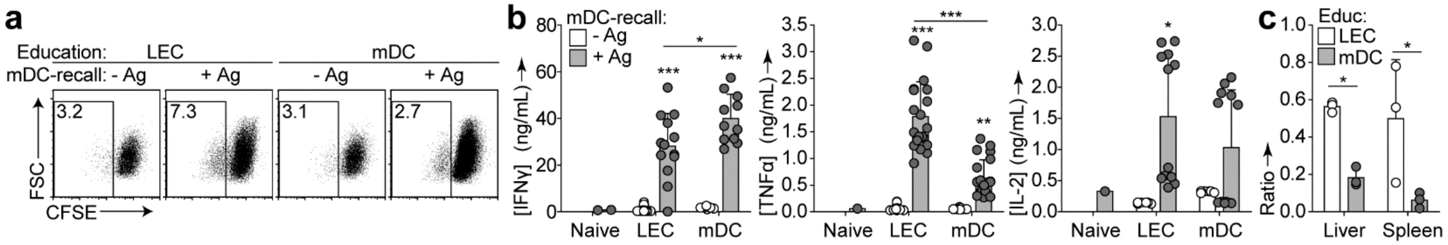

Supplementary Figure 6. Secondary challenge of LEC-educated CD8⁺ T cells creates functional effector cells. Experimental setup for **a-b** follows Fig 7a. CFSE-labeled LEC/mDC-educated cells were re-plated with mDCs (mDC-recall) that had been pulsed with SIINFEKL peptide (+Ag; control unpulsed mDC = -Ag respectively) for 24h. **a** Representative flow cytometry dot plots gated on live OT-I cells, showing size (FSC; forward scatter) versus CFSE dilution. **b** Endpoint concentrations of IFNγ, TNFα, and IL-2 in cell culture supernatants depending on initial education and secondary *in vitro* challenge conditions. Naïve OT-I cells that were received initial stimulation with antigen-pulsed mDCs have been shown for comparison. **c** Experimental schedule and setup follows Fig 7g. LEC- or mDC-educated OT-I cells ($CD45.2^+$) were transferred into C57Bl/6 mice ($CD45.1^+$) and 5 weeks later, mice were treated with OVA+LPS. Ratio of $CD44^+CD62L^+$ to $CD44^+CD62L^-$ at d5 after secondary challenge. Data points and error bars indicate mean ± SD for n=3-21 biological replicates from one representative of at least two independent experiments; *p≤0.05, **≤0.01, ***≤0.001 by one-way (b) or two-way (c) ANOVA with Bonferroni's post-test. For naïve controls in b, n=1 technical replicate was shown for comparison, but was not included for statistical testing.

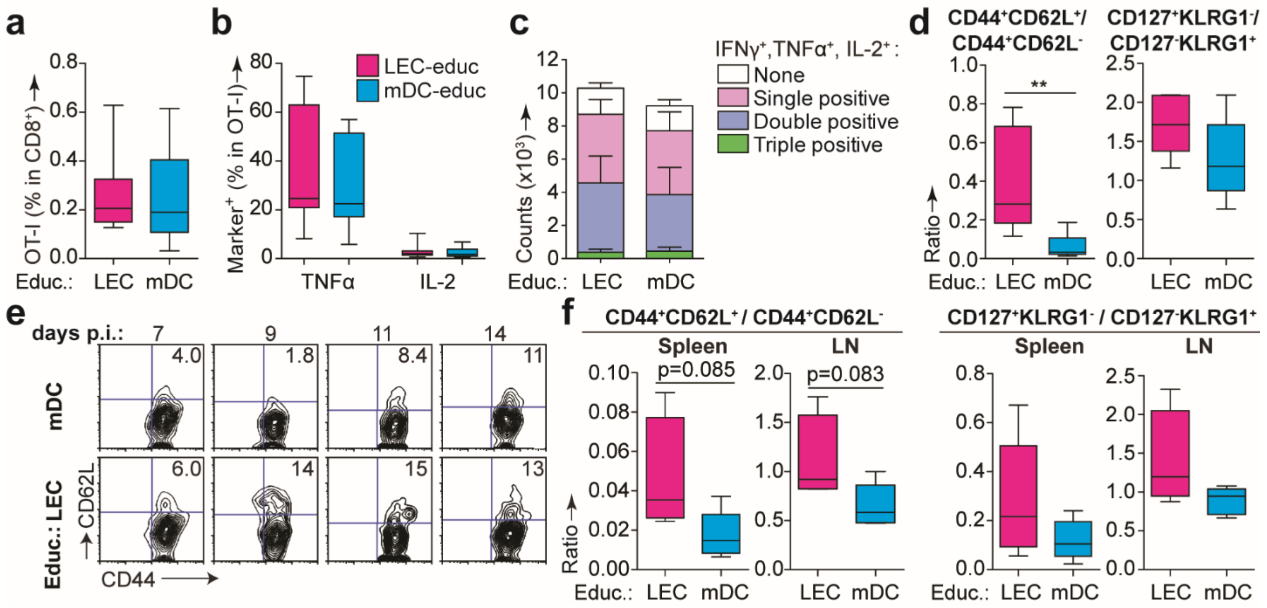

Supplementary Figure 7. LEC-educated CD8⁺ T cells mediate protection against infectious pathogens and maintain a persistent secondary memory population. Experimental setup followed Fig. 8a. $CD45.1/2^+$ LEC-educated OT-I cells were co-transferred with ($CD45.1^+$) mDC-educated cells (1:1) into C57Bl/6 mice, and challenged with *L.m.*-OVA (10^3 cfu/mouse, i.v.) 5w later. **a** Percentage of OT-I cells among all $CD8^+$ T cells detected in skin-draining LNs at d8 post-infection. **b** TNF-a$^+$ and IL-2$^+$ cells (intracellular) as a percentage of splenic OT-I cells at d8 post-infection. **c** Absolute number of recovered splenic OT-I cells that stained positive for at least one of: IFNγ, TNFα, or IL-2. **d** Ratios of memory to effector cells among LEC- vs. mDC-educated OT-I cells recovered from the spleen, using two accepted phenotypic definitions as indicated, at d8 post-infection. **e** Representative plots depicting CD62L and CD44 expression in circulating OT-I cells at different time points following challenge. **f** Ratios of memory to effector cells using two accepted phenotypic definitions as indicated, among LEC- vs. mDC-educated OT-I cells recovered in the spleen and skin-draining LNs at 3w post-infection. (a,b,d,f) Box and whiskers indicate median, min-to-max data ranges; and (c) Data points and error bars depict mean ± SEM for n=5-8 biological replicates pooled from up to two independent experiments. $^{**}p \leq 0.01$ by two-way ANOVA with Bonferroni's post-test (c), or by two-tailed unpaired Student's t-test (a,b,d,f).

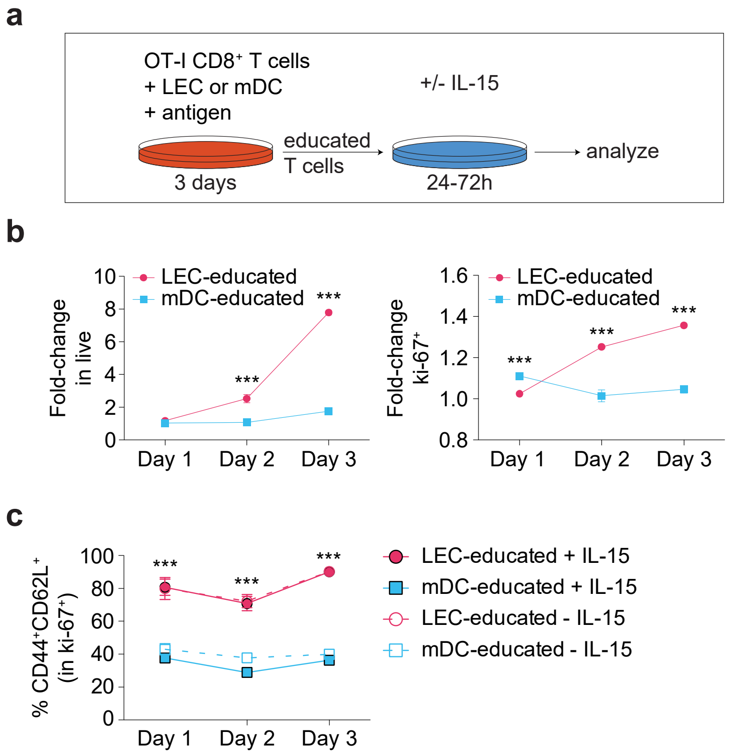

Supplementary Figure 8. LEC-educated CD8⁺ T cells are superior in survival and phenotype maintenance vs. mDC-educated CD8⁺ T cells. a Experimental setup: LEC or mDC-educated OT-I cells (1nM Ag, 3d) were subsequently cultured in the presence or absence of IL-15 for up to 3d before being assessed for viability, proliferation (ki-67 staining) and T_{CM}–like phenotype ($CD44^{+}CD62L^{+}$) by flow cytometry. **b** Fold-change in (left) cell viability and (right) frequency of proliferating (ki-67⁺) cells in IL-15-treated relative to control (same education conditions for first 3d, but no IL-15 for last portion of the schedule). **c** Percentage of $CD44^{+}CD62L^{+}$ cells in LEC- versus mDC-educated cells, depending on presence of IL-15 (filled symbols). Control cultures of OT-I cells that had received same education conditions (empty symbols) are shown for comparison. Note that LEC-educated control (empty magenta) trace follows LEC-educated + IL-15 trace closely. Data points and error bars indicate mean ± SD for n=3 replicates from one independent experiment. ***p≤0.001 by two-way ANOVA with Bonferroni's post-test.

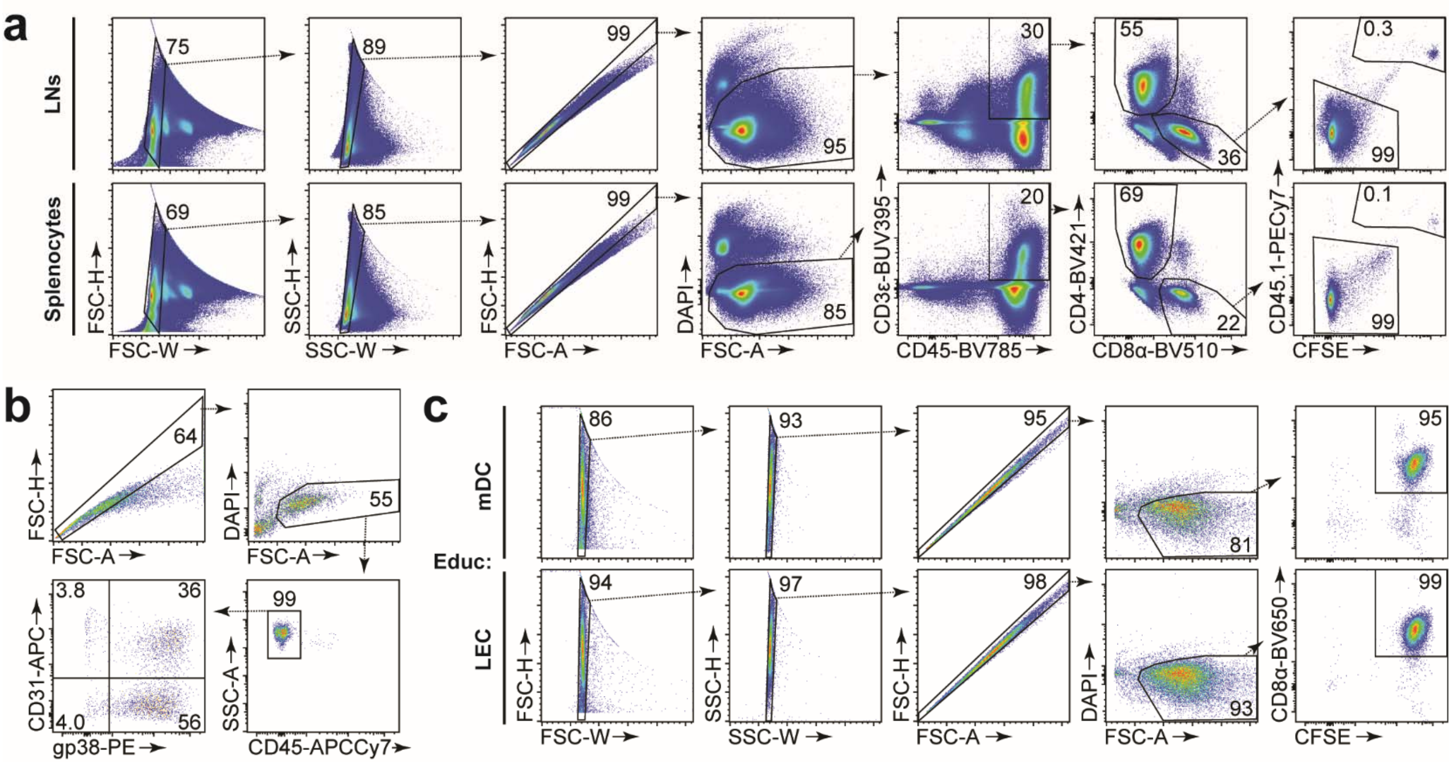

Supplementary Figure 9. Representative flow cytometry gating strategies for experiments in this study. **a** Typical flow cytometry gating strategy for *in vivo* adoptive transfer experiments, such as in Figs. 1,3,6-8. In this example, a splenocyte sample was filtered *in silico* through three serial gates to exclude multiplets, live cells are selected (DAPI vs. FSC-A), and then T cells (CD45$^+$CD3ε$^+$). From here, CD8α$^+$CD4$^-$ T cells were selected, from which OT-I cells were generally positive for a congenic marker (CD45.1$^+$ in this example) as well as a fluorescent cell tracker or proliferation dye for some experiments. **b** Typical flow cytometry sorting strategy to purify for LECs (gp38$^+$CD31$^+$) from among live CD45$^-$ singlet cells from lymph node cell suspensions. **c** Typical flow cytometry gating strategy to identify OT-I cells following co-culture with either LECs or mDCs. This workflow is similar to the one shown for *in vivo* experiments, but typically lacks the use of CD45 and CD3ε stains. All inset numbers indicate percentage of cells that occurred within the designed gates.

Highest in Naive	
	Gene ID:
1	Ighj2
2	Gm15915
3	Gm11695
4	Pydc3
5	Pydc4
6	Gm18752
7	Gm4955
8	Ifi203
9	Gm26740
10	Gm16340
11	Ighd
12	Klf2
13	Cnga1
14	Rtp4
15	Gm18445
16	Irf7
17	Ifit3b
18	AI607873
19	Ifit3
20	E130215H24Rik
21	Art2b
22	Gm14446
23	Gprc5b
24	Art2a-ps
25	A530021J07Rik
26	Slc14a1
27	Baiap3
28	Nsg2
29	Ighm
30	Bambi-ps1
31	Ifit1
32	Timp2
33	2010002M12Rik
34	Gm14086
35	Ddx60
36	Lcn4
37	A930005H10Rik

Highest in LEC-educated	
	Gene ID:
38	Il9r
39	Cd101
40	Ifi27l2a
41	Ifi27l2b
42	Bcl6
43	Gucy1a3
44	Igsf3
45	Nrgn
46	Iigp1
47	Nt5e
48	Gm5970
49	Rasgef1a
50	Gbp6
51	Gm4951
52	Selp
53	Dapl1
54	Isg15
55	Hemgn
56	Gbp6
57	Tspan2
58	Gm5815
59	Arl4d
60	Pde1a
61	Trim30d
62	Gbp10
63	Trim30c
64	Trim30e-ps1
65	Trim30a
66	Trim30b

Highest in mDC-educated	
	Gene ID:
67	Ero1l
68	Gm37881
69	Prdm1
70	Ctcfl
71	Stbd1
72	Ecm1
73	Tmem163
74	Nfil3
75	Egln3
76	Arhgef10
77	Cyp2s1
78	AA467197
79	F730043M19Rik
80	Apln
81	H2-Eb1
82	Stk32c
83	Basp1
84	Aldh1l2
85	Ptrf
86	Gzmg
87	Reep6
88	Filip1
89	Gadd45g
90	6430571L13Rik
91	Zbtb32
92	Myo10
93	Id2
94	P4ha2
95	Dpf1
96	Nrp2
97	Stc2
98	Zfp683
99	Epn2
100	Utf1
101	Gm11714
102	Hic1
103	Nos2
104	Fam20a
105	Bcar1
106	Adm
107	Gm37787
108	Gm6175
109	Nupr1
110	Mt3
111	Slc4a11
112	Nkain1

Highest in mDC-educated	
	Gene ID:
113	Lif
114	Rai14
115	Tnfaip2
116	Rnf208
117	Gm867
118	Gzmf
119	Syt5
120	Gzmd
121	Cdkn1a
122	Gpnmb
123	Dgat2
124	Gatm
125	Pik3r6
126	Il4i1
127	Gja1
128	Serpine1
129	Ccl5
130	Slc41a2
131	Upp1
132	M1ap
133	Lta
134	Il1r2
135	Obsl1
136	Adora2b
137	Atp6v0d2
138	Il23a
139	Guca1a
140	Cish
141	Penk
142	Dntt
143	Rem2
144	Neb
145	Inha
146	Smtnl2
147	Fgf11
148	Sema7a
149	Stc1
150	Osr2
151	Ppp1r3b
152	Acvrl1
153	Trim16
154	Fam183b
155	Il3
156	Lyz2
157	Gcnt4
158	Lyz1

Highest in mDC-educated	
	Gene ID:
159	Lgals3
160	Ttc39c
161	Mt1
162	P2rx7
163	Gm14668
164	Asb16
165	F3
166	Ifng
167	Batf3
168	Fcer1g
169	Pla2g7
170	Lama5
171	Serpinf1
172	Sncb
173	Ccr5
174	Ccl9
175	Ermn
176	Csf2rb
177	Mt2
178	Gm16350
179	Gp49a
180	Csf2rb2
181	Sox5os3
182	Fscn1
183	Crabp2
184	Emilin2
185	Adam8
186	Gpr15
187	Clec7a
188	Ccl6
189	Adamts14
190	Ccr1
191	Gzma
192	Inhba
193	Gpr141
194	Fgfbp1
195	Mmp12
196	Tgm1
197	Asb2
198	Il21
199	H2-M2
200	Coch

Supplementary Table 1. The 200 most differentially expressed genes between d3 LEC- versus mDC-educated $CD8^+$ T cells, which were also significantly modulated relative to naïve OT-I cells ($|log_2FC|>2.89$, adj. FDR-adjusted $p < 0.01$).

Supplementary Methods

Generation of bone marrow chimeras

Bone marrow (BM) was recovered from tibiae and femurs by flushing with PBS-EDTA and dissociated by repeated passages through a 20-gauge needle. To generate bone marrow chimeras, recipient mice (C57BL/6 wild-type or $\beta_2m^{-/-}$ mice) were gamma-irradiated twice with 450 rad (4h apart) and reconstituted by tail vein injection with $8x10^6$ bone marrow cells from wild-type or $\beta_2m^{-/-}$ donor mice. Chimeras were rested for 8 weeks and, following confirmation of reconstitution by flow cytometry of blood cells, they were subsequently enrolled in experiments.

Adoptive Transfers of CD8$^+$ T cells

For bone marrow chimera experiments, 10^6 purified naïve OT-I CD8$^+$ T cells (CD45.1/2) were adoptively transferred intravenously by tail vein injection in 100 µl IMDM (Life Technologies, Carlsbad, CA, USA) into chimeric mice (CD45.2). To determine the homing potential of LN LEC/mDC-educated CD8$^+$ T cells, 10^6 cells (CD45.1) were adoptively transferred in naïve host mice (CD45.2). For the *in vivo* antigen re-encounter experiments, 10^6 LEC/mDC-educated cells (CD45.2) were adoptively transferred in naïve host mice (CD45.1). For the studies including challenge with bacterial pathogen, $5x10^4$ LN LEC/mDC-educated CD8$^+$ T cells (CD45.1 or CD45.1/2) were adoptively transferred in naïve host mice (CD45.2).

Organ collection and processing into single cell suspensions.

Spleens, LNs (brachial, axillary, inguinal, popliteal), as well as lungs, liver and BM when indicated, were harvested at time of killing. LNs were digested 45min, in DMEM supplemented with 0.15 Wünsch Units/ml collagenase D (Roche, Basel, CH). Single-cell suspensions were obtained by gently disrupting the spleen and LNs through a 70-µm cell strainer. BM was recovered from tibia and femurs by flushing with medium through a 20-gauge needle and passed through a 70-µm cell strainer. Lungs were perfused with 10ml PBS, digested in medium with collagenase D for 45 min and the remaining tissue disrupted as described above. Afterwards, a 30% Percoll (VWR, Dietikon, Switzerland) gradient was applied to the cells to isolate lung leukocytes. Liver was perfused with 10ml PBS and gently disrupted through a 100-µm and subsequently, 70-µm cell strainer. A 37.5% Percoll gradient was then applied to the cells to isolate liver leukocytes. Spleen, BM, liver and blood RBCs were lysed with NH_4Cl for 4 min. Cells were counted and resuspended in co-culture media.

Ex vivo re-stimulation

Up to $3x10^6$ cells were plated in 96-well plates and cultured in co-culture media for 2 hours at 37°C in the presence of 1 µg/mL SIINFEKL peptide, followed by additional 3h treatment with BFA (5 µg/mL). Stimulation with PMA/ionomycin served as a control. For CD107a staining, the monoclonal antibody against CD107a was added in the culture together with monensin at 5 µg/mL for 5 hours. Finally, cells were washed in PBS prior to intracellular staining for flow cytometric analysis.

LN-Homing and Localization by Immunofluorescence

To determine the specific localization of LEC-educated CD8$^+$ T cells in the LN, we adoptively co-transferred naïve and LEC-educated CD8$^+$ T cells at a ratio of 1:1 ($5x10^6$ total cells). Prior to transfer, naïve cells were labeled with CFSE and LEC-educated CD8$^+$ T cells with eFluor670 Cell Proliferation Dye (eBioscience). 24-48h later, brachial LNs were removed and fixed overnight in 2% PFA in PBS pH 7.4. After 3 washes in PBS, LNs were embedded in a block of 2% agarose, and sectioned (150µm) using a using a vibratome (Leica, Wetzlar DE). Sections were blocked in 0.5% of casein, and further labeled using antibodies against B220 (Invitrogen, Auckland, NZ, USA) and LYVE1 (Reliatech, San Pablo, CA US). Images were acquired on a Leica SP5 confocal microscope using 20x or 60x objectives, and processed using Imaris software (Bitplane, Zürich, CH).

Cytokine detection

Ready-SET-go! ELISA kits for cytokine detection were purchased from eBioscience, except for the IL-7 mouse ELISA kit (Abcam, Cambridge, UK), and used according to the manufacturers' instructions. For multiple cytokine detection, a custom 37-plex Luminex assay (eBioscience) was performed according to the manufacturers' instructions.

In vitro homeostatic proliferation

To determine whether LEC-educated CD8$^+$ T cells can proliferate in response to homeostatic signals, we exposed them to IL-15 for different time periods. LEC/mDC-educated CD8$^+$ T cells were harvested on day 3 of co-culture, washed at least twice with basal medium and counted. 10^5 LEC-educated or mDC-educated CD8$^+$ T cells were subsequently cultured in the presence or absence of IL-15 (20 U/ml). 24-72h later, the cells were harvested and stained for immunological markers to be analyzed by flow cytometry.
