# Supplementary material for: Lymphatic endothelial cells prime naïve CD8+ T cells into memory cells under steady-state conditions
Source: Nat Commun. 2020 Jan 27;11:538. doi: 10.1038/s41467-019-14127-9 (PMC6985113; doi:10.1038/s41467-019-14127-9)
Supplement: Supplementary file 2 — Reporting Summary [file 41467_2019_14127_MOESM2_ESM.pdf]

nature research

Corresponding author(s): Melody A. Swartz
Last updated by author(s): Dec 16, 2019

Reporting Summary

Nature Research wishes to improve the reproducibility of the work that we publish. This form provides structure for consistency and transparency in reporting. For further information on Nature Research policies, see Authors & Referees and the Editorial Policy Checklist .

Statistics

For all statistical analyses, confirm that the following items are present in the figure legend, table legend, main text, or Methods section.

n/a	Confirmed	
☐	☒	The exact sample size (*n*) for each experimental group/condition, given as a discrete number and unit of measurement
☐	☒	A statement on whether measurements were taken from distinct samples or whether the same sample was measured repeatedly
☐	☒	The statistical test(s) used AND whether they are one- or two-sided<br>*Only common tests should be described solely by name; describe more complex techniques in the Methods section.*
☒	☐	A description of all covariates tested
☐	☒	A description of any assumptions or corrections, such as tests of normality and adjustment for multiple comparisons
☐	☒	A full description of the statistical parameters including central tendency (e.g. means) or other basic estimates (e.g. regression coefficient) AND variation (e.g. standard deviation) or associated estimates of uncertainty (e.g. confidence intervals)
☐	☒	For null hypothesis testing, the test statistic (e.g. *F*, *t*, *r*) with confidence intervals, effect sizes, degrees of freedom and *P* value noted<br>*Give P values as exact values whenever suitable.*
☒	☐	For Bayesian analysis, information on the choice of priors and Markov chain Monte Carlo settings
☐	☒	For hierarchical and complex designs, identification of the appropriate level for tests and full reporting of outcomes
☒	☐	Estimates of effect sizes (e.g. Cohen's *d*, Pearson's *r*), indicating how they were calculated

Our web collection on statistics for biologists contains articles on many of the points above.

Software and code

Policy information about availability of computer code

Data collection	Flow cytometry collection via FACSDiva 6.1, Leica LAS X for microscopy or Zeiss ZEN 2010, RNASeq data collection using the Illumina software
Data analysis	GraphPad Prism v5, FlowJo version X, all methods for RNAseq analysis within the Transcriptional profile analysis section within Methods

For manuscripts utilizing custom algorithms or software that are central to the research but not yet described in published literature, software must be made available to editors/reviewers. We strongly encourage code deposition in a community repository (e.g. GitHub). See the Nature Research guidelines for submitting code & software for further information.

Data

Policy information about availability of data

All manuscripts must include a data availability statement. This statement should provide the following information, where applicable:

- Accession codes, unique identifiers, or web links for publicly available datasets
- A list of figures that have associated raw data
- A description of any restrictions on data availability

The RNA sequencing data have been deposited to the NIH Gene Expression Omnibus under accession number GSE88830. Additional data has been referenced in the Supplementary Information, and raw data for all figures and supplementary figures are available in the Source Data file. Any additional data files are available from the authors upon reasonable request.

Field-specific reporting

Please select the one below that is the best fit for your research. If you are not sure, read the appropriate sections before making your selection.

Life sciences study design

All studies must disclose on these points even when the disclosure is negative.

Sample size	The number of animals chosen per experiment was based on previous publications using similar models and addressing similar questions, as well as on previous studies performed in our lab when statistical significance was actually achieved. To account for data reproducibility, the experiments were performed at least twice. (Online Methods/ Statistical analysis section)
Data exclusions	Animals were excluded from flow cytometric analysis only in case the number of events was extremely low, generating outliers or false-positive data.
Replication	Generally, expleriments were repeated 2-3x. In the RNAseq experiment, the costs were prohibitive enough to prevent replication of this study, so this data was based off one experimental repeat. However, the setup of the RNAseq study matches the setup used in the flow cytometry studies in Figures 2-3 - just that the method of data acquisition/characterization was different.
Randomization	In all the animal studies, mice were randomly assigned to the different groups and treated in a random order. Individual cages included mice across multiple groups in order to reduce the likelihood of cage effects.
Blinding	The investigators were not blinded to group allocation during experiment setup or data analysis. However, in between the setup and the actual endpoint (i.e., while the animals were alive), since individual cages contain mice across multiple treatment groups, investigators were partially blind to which mouse belonged to which treatment group, especially when performing daily or routine checks for pain/suffering/body condition according to ethical guidelines. Bleeding and organ collections/harvest were often collected in containers that at the very least, contained mouse ID#, but the actual treatments/experimental conditions that corresponded to the individual ID#s were kept by the same investigators in lab notebooks that were not always within immediate reach until the data analysis stage.

Reporting for specific materials, systems and methods

We require information from authors about some types of materials, experimental systems and methods used in many studies. Here, indicate whether each material, system or method listed is relevant to your study. If you are not sure if a list item applies to your research, read the appropriate section before selecting a response.

Materials & experimental systems

n/a	Involved in the study	
☐	☒	Antibodies
☒	☐	Eukaryotic cell lines
☒	☐	Palaeontology
☐	☒	Animals and other organisms
☒	☐	Human research participants
☒	☐	Clinical data

Methods

n/a	Involved in the study	
☒	☐	ChIP-seq
☐	☒	Flow cytometry
☒	☐	MRI-based neuroimaging

Antibodies

Antibodies used	Detailed in Methods / Reagents section & Flow cytometry sections.
Validation	The following anti-mouse antibodies were used: CD62L (MEL-14), CD44 (IM7), KLRG1 (2F1/KLRG1), PD-1 (RMP1-30), CXCR3 (CXCR3-173), CD43 (1B11), CD27 (LG.3A10), CD122 (TM-b1), CCR7 (4B12), CD127 (A7R34), T-bet (eBio4B10), Eomes (Dan11mag), LFA-1 (H155-78), Sca-1 (D7), Bcl-2 (BCL/10C4), CD45.1 (F20), CD45.2 (104), CD8 (53-6.7), CD3e (145-2C11), IFNγ (XMG1.2), IL-2 (JES6-5H4), TNFα (MP6-XT22), CD107 (1D4B), Granzyme-B (NGZB), ki-67 (SolA15), pS6S235/236 (D57.2.2E), pmTORS2448 (O21-404), pAktS473 (D9E). Phospho-flow antibodies were from Cell Signaling Technology (Danvers, MA, USA). Validation statements are available on the manufacturers' websites (BioLegend, eBioscience, BD Biosciences).

Animals and other organisms

Policy information about studies involving animals; ARRIVE guidelines recommended for reporting animal research

Laboratory animals	Methods, Mice section
Wild animals	None
Field-collected samples	None
Ethics oversight	All procedures were approved by the Cantonal Veterinary Committee of Vaud, Switzerland (VD2518 and VD2992) or the University of Chicago IACUC (protocol # 72414 & 72578).

Note that full information on the approval of the study protocol must also be provided in the manuscript.

Flow Cytometry

Plots

Confirm that:

- [ ] The axis labels state the marker and fluorochrome used (e.g. CD4-FITC).
- [x] The axis scales are clearly visible. Include numbers along axes only for bottom left plot of group (a 'group' is an analysis of identical markers).
- [x] All plots are contour plots with outliers or pseudocolor plots.
- [x] A numerical value for number of cells or percentage (with statistics) is provided.

Methodology

Sample preparation	Methods - Flow Cytometry Section
Instrument	CyAn ADP Flow Cytometer (DAKO), BD LSR Fortessa, BD LSR-II, BD FACSAria IIu, BD FACSAria IIIu, BD FACSFusion
Software	FACSDiva 6.1
Cell population abundance	50,000 - 200,000 LN-LECs (>99%, determined by the sorting cytometer)<br>50,000 - 200,000 LN-FRCs (>99%, determined by the sorting cytometer)<br>>1,000,000 DCs (>99%, determined by the sorting cytometer)<br>>50,000 macrophages (>99%, determined by the sorting cytometer)<br>5-10 million naive OT-I cells/donor mouse (>99%, determined by the sorting cytometer)<br><5 million CD44+ OT-I cells/donor mouse (>99%, determined by the sorting cytometer)
Gating strategy	See Supplementary Figure 9.

- [x] Tick this box to confirm that a figure exemplifying the gating strategy is provided in the Supplementary Information.
